# RAFFI: Accurate and fast familial relationship inference in large scale biobank studies using RaPID

**DOI:** 10.1371/journal.pgen.1009315

**Published:** 2021-01-21

**Authors:** Ardalan Naseri, Junjie Shi, Xihong Lin, Shaojie Zhang, Degui Zhi

**Affiliations:** 1 School of Biomedical Informatics, The University of Texas Health Science Center at Houston, Houston, Texas, United States of America; 2 Department of Computer Science, Rice University, Houston, Texas, United States of America; 3 Department of Biostatistics, Harvard T. H. Chan School of Public Health, Boston, Massachusetts, United States of America; 4 Program in Medical and Population Genetics, Broad Institute of Harvard and MIT, Cambridge, Massachusetts, United States of America; 5 Department of Statistics, Harvard University, Cambridge, Massachusetts, United States of America; 6 Department of Computer Science, University of Central Florida, Orlando, Florida, United States of America; 7 Center for Precision Health, The University of Texas Health Science Center at Houston, Houston, Texas, United States of America; University of Washington, UNITED STATES

## Abstract

Inference of relationships from whole-genome genetic data of a cohort is a crucial prerequisite for genome-wide association studies. Typically, relationships are inferred by computing the kinship coefficients (*ϕ*) and the genome-wide probability of zero IBD sharing (*π*_0_) among all pairs of individuals. Current leading methods are based on pairwise comparisons, which may not scale up to very large cohorts (e.g., sample size >1 million). Here, we propose an efficient relationship inference method, RAFFI. RAFFI leverages the efficient RaPID method to call IBD segments first, then estimate the *ϕ* and *π*_0_ from detected IBD segments. This inference is achieved by a data-driven approach that adjusts the estimation based on phasing quality and genotyping quality. Using simulations, we showed that RAFFI is robust against phasing/genotyping errors, admix events, and varying marker densities, and achieves higher accuracy compared to KING, the current leading method, especially for more distant relatives. When applied to the phased UK Biobank data with ~500K individuals, RAFFI is approximately 18 times faster than KING. We expect RAFFI will offer fast and accurate relatedness inference for even larger cohorts.

## Author summary

Inferring familial relationships has a wide range of applications. Family-based genome-wide association studies and population-based GWAS both require genetic relationships. Inferring relationship is essential for unknown familial structures and can be used to correct pedigree information due to false paternity, sample switches, or unregistered adoption. Current approaches for inferring relationships are not scalable for large cohorts comprising millions of individuals. Here, we present a fast and flexible method, called RAFFI, using Identical by Descent (IBD) segments. IBD segments are uninterrupted DNA segments inherited from a common ancestor. Relationships are usually inferred by computing the kinship coefficients and the genome-wide probability of zero IBD sharing among all pairs of individuals. In the first step, we search for IBD segments using RaPID which avoids a pairwise comparison of all individuals in a haplotype panel. In the second step, we compute the kinship coefficients to infer the relationships. To make our method robust against genotyping and phasing error, the thresholds of kinship coefficients for different degrees of relatedness are adjusted. As a result, the lower detection power of IBD segments due to phasing errors or misspecification of the genotyping error rate will not comprise the inference of relationships.

## Introduction

Inference of hidden/cryptic relatedness in large genetic cohorts is often a prerequisite for successful genome-wide association studies (GWAS). For example, for family-based genome-wide association studies (GWAS), complete and accurate information about the relatedness is necessary for proper adjustment of familial random effects. For population-based GWAS, mixed effect models are also increasingly used by explicit use of dense genotype relationship matrix (GRM) [1–3]. While GRM can be inferred from the whole genome genotypes, identifying relatedness (e.g., 3^rd^ or 4^th^ degree relatives) can provide a sparse GRM for which efficient association algorithms could be derived. Moreover, even well-annotated cohorts may still contain incorrect pedigree information due to falsely claimed paternity, sample switches, or unregistered/unknown adoption. In such cases, inferred relatedness from genotype data can be used for checking and correction of pedigree information [4] and sample quality control [5].

Recent advances in SNP array genotyping and whole-genome sequencing have led to the generation of abundant genotype data. Current biobank data—such as UK Biobank [6], All of Us Research Project [7], or Million Veteran Project [8], comprise hundreds of thousands up to millions of individuals. The availability of large cohorts of genetic data increases the power for association studies and enables studies of fine-scale population history. At the same time, it brings a challenge to efficiently utilize the data. A major issue in the inference of relatedness in large cohorts, as more and more genotype data become available, is computational efficiency. Current practice typically relies on pairwise comparisons of all individuals in a panel, resulting in quadratic computational complexity. Scaling up such an approach to large biobanks will require extensive and costly resources that are not practical.

Identical by Descent (IBD) is a fundamental concept in genetics and inheritance. IBD segments are defined as DNA segments that have been passed from a common ancestor [9]. More recent common ancestors will typically result in longer IBD segments, and thus IBD segments are informative for inferring familial relationships. IBD segment-based inference methods [10–12] are believed to be more accurate, as they are less vulnerable to elevated global genotype similarity in admixed populations. Nevertheless, non-IBD based inference methods [13,14] are thought to be faster and have been dominating the field of relatedness inference for large cohorts. For example, KING has been the most commonly used for estimating kinship [13]. However, KING is based on pairwise comparisons with genome-wide genotypic similarity and does not scale up to very large cohorts (e.g., >1 million samples). Moreover, to achieve efficiency in very large cohorts, KING applies some fast genotype similarity filters and results in loss of sensitivity for slightly distant relatives (e.g., 3^rd^ or 4^th^ degree relatives). Therefore, relatedness inference over large cohorts with a complex population structure remains a challenging problem.

Recently, efficient IBD segmental detection algorithms that avoid pairwise comparison [15–18] for large cohorts have been developed. Although seemingly paradoxical, in large cohorts, identifying all pairwise long segment matches can be more efficient than computing all pairwise overall genotype similarities. This is because the former has a linear time algorithm [19] while the latter needs quadratic time algorithms. Among fast IBD segment detection methods, hash table-based methods [16,17] are typically memory intensive. RaPID [15] and hap-IBD [18] are based on the scanning algorithm of PBWT and are scaling up both in terms of run time and memory. hap-IBD used a seed-and-extension approach and was tuned for identifying short IBD segments with high accuracy but sacrifices detection power [18]. RaPID, instead, has a principled yet flexible statistical framework for achieving a balance between accuracy and detection power. Therefore, in this work, we choose RaPID to investigate whether IBD segment-based methods will offer fast and yet accurate relatedness inference in very large cohorts. To the best of our knowledge, our method is the first IBD-based approach for relatedness inference in large biobank-scale cohorts.

We developed a new IBD-based method called RAFFI for efficient relatedness inference in phased haplotypes of human cohorts. RAFFI provides a more efficient approach with regards to run time and also proves to be robust in inferring relatedness. We conducted extensive simulation studies to evaluate the efficiency and accuracy of RAFFI and the current leading method, KING. Despite the efficiency of KING, it may not guarantee a linear run time which would be problematic for studies comprising hundreds of thousands or millions of individuals, such as in biobanks. The run time of RAFFI is dependent on the detected IBD segments by RaPID which guarantees a linear run time for long segments (e.g. 5 cM). Moreover, we introduced a new data-driven approach for robust inference for degrees of relatedness in the presence of genotyping/phasing errors in real data sets. While KING is somehow robust against limited genotyping errors, it does not have the flexibility to be easily adjusted in studies with known/unknown high or low genotyping errors or population structure. The results of KING may be affected by the genotyping error rate, which may vary between pairs included in homozygous or heterozygous populations, or by the presence of admixed individuals that have different background heterozygous rates. We specifically investigated the robustness of these methods in the presence of phasing and genotyping errors, multi-way admixture, and variable marker densities. We also evaluated the efficiency of the methods in real data of UK Biobank.

## Materials and methods

### Overview of the RAFFI method

The RAFFI method has three steps. In the first step, we search for IBD segments among all pairs of individuals in a study using RaPID. The input data for detecting IBD segments and inferring relatedness are phased haplotypes. In the second step, we calculate the raw kinship coefficients (***ϕ*** and ***π***_**2**_, the fraction of the genome that is IBD2, i.e., IBD in both copies of the genome) among all pairs sharing IBD segments. In the final step, we estimate the effect of phasing/genotyping error on the kinship values inferred by the IBD segments, adjust the kinship values for inference of relatedness, and report the related individuals.

### IBD segment detection

There are several fast methods for IBD segment detection available [15,17,18]. Most methods are based on the seed-and-extension framework: While optimized for the identification of individual IBD segments accurately, these methods are typically biased towards accuracy but sacrificing detection power. RaPID is based on multiple low-resolution random projections of the original study: It offers theoretically optimal run time complexity and is more flexible for adjusting the balance between detection power and accuracy. Therefore, we choose RaPID for the IBD detection step.

The overall probabilistic framework of RaPID was described in [15]. Briefly, for an IBD segment of *S* cM, we first pick a random SNP out of a window of *w* cM, and make the IBD segment call if each of a consecutive *L* = *S*/*w* windows returns an exact match between two haplotypes. We estimate the probabilities of true positive and false positive as follows. Assuming a mismatch rate of *ϵ* for the sum of both mutation rate and genotyping error rate, the binomial probability in each run is (1−*ϵ*)^*L*^≈*e*^−*Lϵ*^. The probability calculation can be boosted by taking *r* random rounds, that the segment is covered by any of the *r* rounds is:

$$t_{p}=1-(1-e^{-L\epsilon }{)}^{r}.$$

Similarly, the probability of having a false hit with the minimum length of *L*-window is *ρL*, where *ρ* is the probability that a randomly chosen pair of individuals would share the projected sequencing in a window. The parameter *ρ* is calculated by scanning the alleles in the panel. The probability of false positives among the *r* rounds is:

$$f_{p}=1-(1-\rho^{L}{)}^{r}.$$

To control false positive one can require at least *c* successes out of *r* runs. For that, *t*_*p*_ and *f*_*p*_ can be calculated by the binomial formula. The objective is that, for given *ρ*, *ϵ*, *S*, we can identify parameter *w*, *r*, *c*, such that *t*_*p*_≈1 and *f*_*p*_≈0. As we showed in the Materials and Methods section “Determination of parameters” subsection of [15], solving this optimization problem often gives a wide range of acceptable parameter choices that can achieve the desired power and accuracy: one can simply pick one in the middle of the range.

By default, we search for IBDs with a length of more than 5 cM (*S* = 5). In the original RaPID we typically set the number of runs *r* = 10 and require at least *c* = 2 out of these runs the IBD segments were identified. However, for relatedness inference purposes, we do not aim to optimize the accuracy of each IBD segment, but rather aim at detecting global parameters relevant to the estimation of kinship coefficients. In RAFFI, we choose *r* = 3 and *c* = 1. We choose a low *r* because it is more efficient while maintaining reasonable estimates of global kinship calculation. Specifically, three runs (r = 3) and only one success (c = 1) would result in high detection power as shown in [15] (see S2 Fig in [15]) while maintaining relatively high accuracy.

### Kinship coefficient calculation

Kinship coefficients are calculated among pairs sharing at least one IBD segment, which is typically quite sparse for samples from outbred populations. First, the IBD segments are separated into IBD1 and IBD2 segments. IBD1 segments are haploid matches between any pair of individuals where only a pair of haplotypes are involved. IBD2 segments are diploid matches where both haplotypes of a pair of individuals match, more specifically both haplotype matches were inherited from common ancestor(s). Following a similar decision-making process of KING that uses the kinship coefficient (*ϕ*) and the fraction of IBD0 segments (*π*_0_), we also calculate these quantities but by using a data-driven approach.

**Fig 1:**
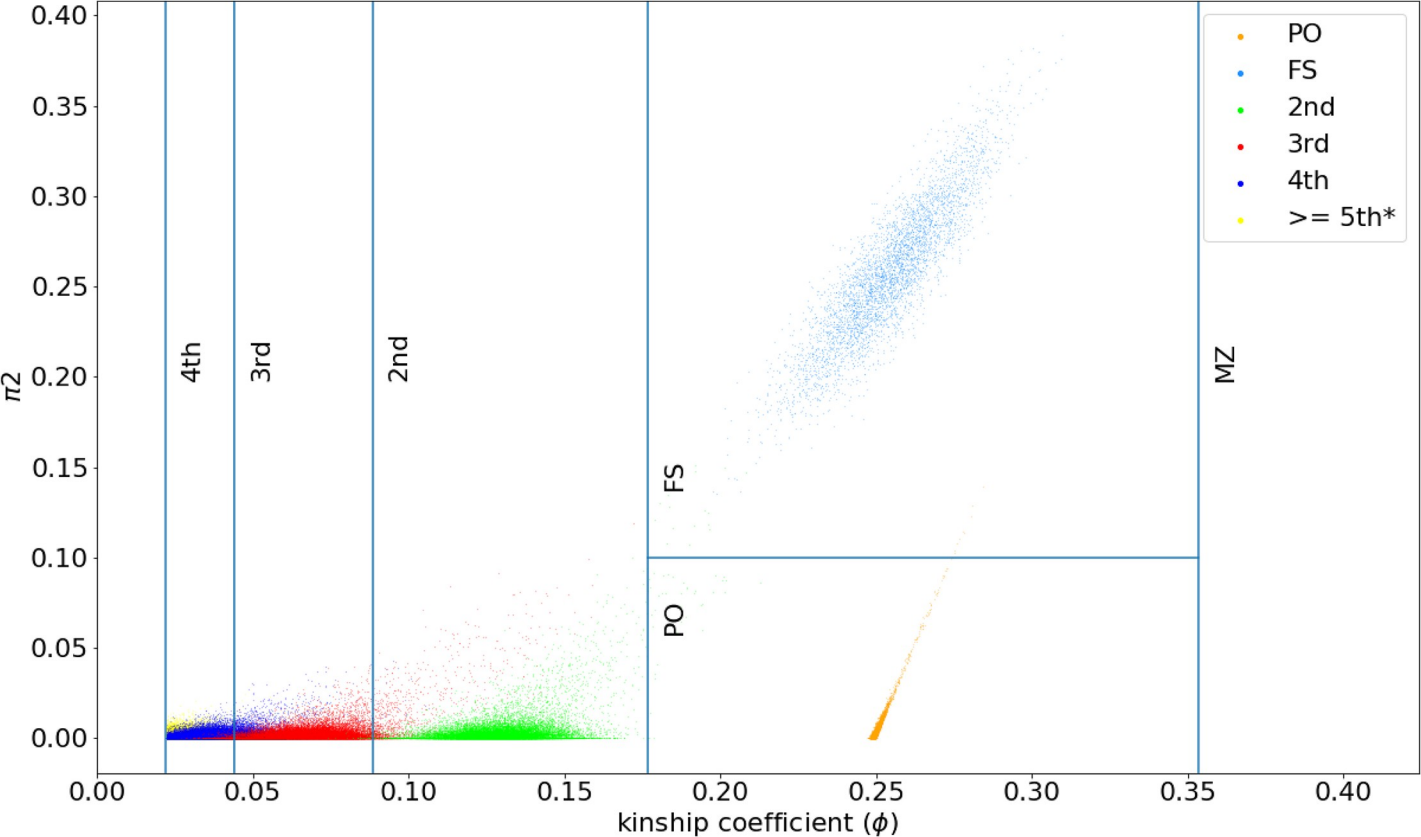
Kinship coefficients computed by RaPID using IBD segments can separate different degrees of relatedness. Kinship coefficients are computed by the total sum of IBDs from RaPID results among pairs with different degrees of relatedness data in simulated data. Different degrees of relatedness (up to 4^th^ degree) can be easily distinguished using the kinship coefficients.

Table 1 contains the expected kinship coefficient and the threshold cutoffs for inferring different degrees of relatedness, following KING’s decision boundaries. Table 2 shows the threshold cutoff for separating parent/offspring and full-sibling pairs using IBD2 segments. Using simulated data (see **Simulated datasets** subsection), we verified that the expected decision boundaries for different degrees of relatedness up to 4^th^ degree are consistent with computed kinship coefficients from RAFFI (**Fig 1**).

**Table 1:** Inference criteria using kinship coefficients [13].

Relationship	*ϕ* Expected	Cutoff
**MZ twin**	1/2	(1/2^3/2^, 1/2^1/2^)
**Parent/offspring**	1/4	(1/2^5/2^, 1/2^3/2^)
**Full-sibling**	1/4	(1/2^5/2^, 1/2^3/2^)
**2^nd^ degree**	1/8	(1/2^7/2^, 1/2^5/2^)
**3^rd^ degree**	1/16	(1/2^9/2^, 1/2^7/2^)
**4^th^ degree**	1/32	(1/2^11/2^, 1/2^9/2^)
**Unrelated**	0	<1/2^11/2^

**Table 2:** Inference criteria for separating parent/offspring and full-sibling pairs using IBD2 segments.

Relationship	*π*_2_ Expected	Cutoff
**Parent/offspring**	0	< 0.1
**Full-sibling**	1/4	> = 0.1

**Fig 2:**
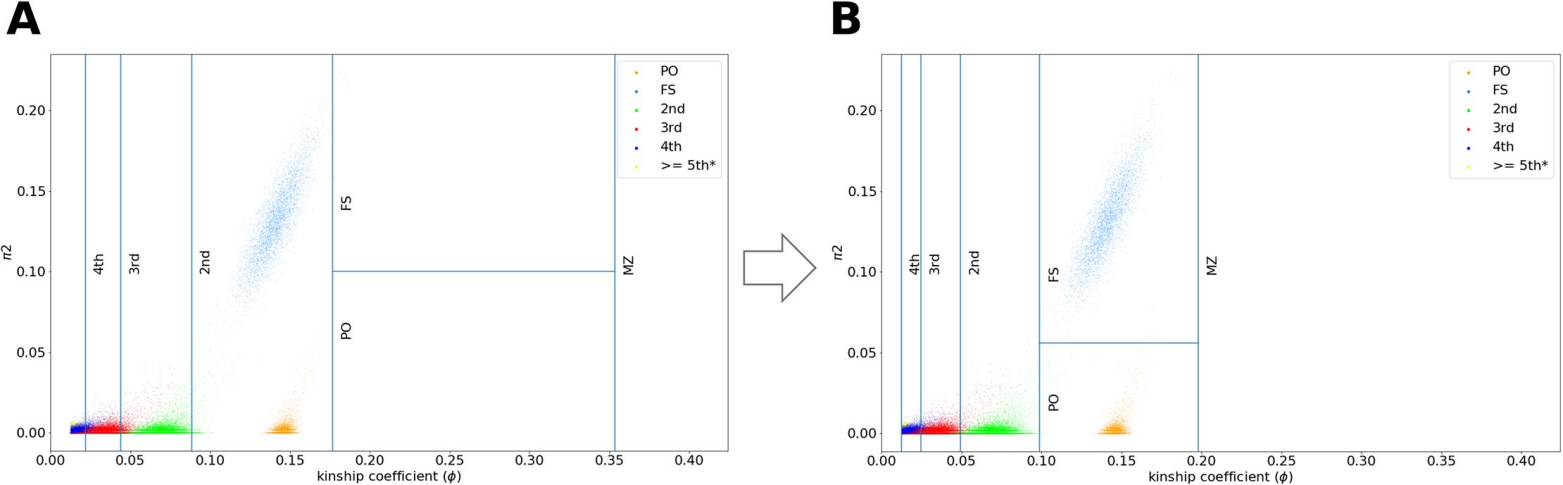
Kinship coefficient thresholds to infer the degrees of relatedness. Kinship coefficients computed by the total sum of IBDs using RaPID in a dataset with phasing and genotyping errors with (**a**) the expected kinship coefficient thresholds, and (**b**) adjusted kinship coefficient thresholds for different degrees of relatedness accounting for phasing/genotyping errors.

The main reason we adopt the data-driven approach is that due to imperfections of haplotype phasing, the lengths of the detected IBD1 and IBD2 segments might be shorter than their real length. As a result, the IBD segments between even very close relatives such as parent-offspring or full-siblings may not extend to their expected length. We observe that phasing errors affect the lengths of IBD segments approximately proportionally (**Fig 2A**, to be detailed in the next sections). Based on this observation, we introduce an adjustment factor *α* as the fraction of the full IBD segments that are detectable (see the next section on how to estimate *α*). For relatedness estimates, we first calculate the raw values of the kinship coefficient (*ϕ*) and the fraction of IBD2 segments (*π*_2_):

$$\phi_{raw}=IBD1/4L+IBD2/2L,\;\mathrm{and}$$ (1)

$$\pi_{2,raw}=IBD2/L,$$ (2)

where *IBD*1 denotes the length of the genome covered by IBD1 segments and *IBD*2 denotes the length of the genome covered by IBD2 segments, and *L* denotes the total length of the genome.

We then calculate the adjusted kinship coefficient (*ϕ*^*α*^) and the fraction of IBD2 segments (*π*_2_^*α*^) as the estimates of the true *ϕ* and *π*_2_ values in the presence of phasing errors:

$$\phi^{\alpha }=\phi_{raw}/\alpha ,\;\mathrm{and}$$ (3)

$$\pi_{2}{}^{\alpha }=\pi_{2,raw}/\alpha .$$ (4)

### Estimating adjustment factor *α* of kinship coefficients

As widely known, the power of IBD segment calls from haplotypes is affected by phasing errors. While phasing error rates (major switch error rate) in large high-quality biobanks are small—about 1 in every 20 cM [20,21] in data sets like UK Biobank, phasing error rate can be higher in smaller cohorts. Also, phasing error rates can be higher in some minority individuals in biobanks and thus still shorten the total length of IBD segments detected.

To evaluate the effect of phasing errors on the lengths of IBD segments detected by RaPID, we simulated random phasing errors (~3 in every 20 cM) while searching for IBD segments with a minimum length of 5 cM. Indeed, we observed an overall proportional reduction of *ϕ* and *π*_2_ (**Fig 2A**). Interestingly, the pairs with high *π*_2_ values, presumably full siblings, still maintain higher *π*_2_ values compared to other pairs. This observation leads to the following idea to estimate *α*, the adjustment factor.

If we know *F* the set of full sibling pairs, we can simply have that their average raw kinship values should be *E*_*F*_(*ϕ*_*raw*_) = α*0.25, where 0.25 is the expected kinship coefficient between a pair of full siblings, and thus can derive

$$\alpha =E_{F}\left(\phi_{raw}\right)/0.25.$$ (5)

However, the set *F* is not known for a typical biobank cohort. If we know *α*, according to KING’s decision boundary, we have:

$$F=\{p|\pi_{2,raw}(p)>\alpha *0.1\}.$$ (6)

In reality, neither *F* nor *α* is known. But Eqs (5) and (6) suggest that the optimal value of *α* is the stationary point of an iterative algorithm. To solve it, we initialize the set *F* to be the top *k*_0_ (default 50) pairs with highest *π*_2,*raw*_ values, and then iteratively apply Eqs (5) and (6) in turn. The iterations are halted as soon as the maximum number of required full-sibling pairs for estimating the factor (by default 1000) is reached or no more than a certain number of full-sibling pairs can be added (by default 50). The pseudocode of this algorithm is available in **S1 Appendix**. As shown in **Fig 2B**, such adjustment of decision boundaries is sufficient for rescuing the loss of detection power of IBD segments due to genotyping/phasing errors. The loss of IBD detection power is observed across all degrees of relatedness (see **S1 Fig**). The adjustment factor *α* here is estimated to be 0.56. If more phasing errors are available then the adjust factor will be even smaller. As a result, the kinship coefficient boundary for 1^st^ degree relatives is shifted from 0.1 to 0.56 and other thresholds are also adjusted accordingly (e.g. 4^th^ degree threshold is reduced from 0.022 to 0.0123).

The above calculation assumes a sufficiently large set of full-sib pairs are known. This assumption is not difficult to satisfy in a large biobank encompassing a substantial fraction of a large population. E.g., in UK Biobank, 22,667 full-sib pairs were found [6]. If not enough full-sib pairs are available, pairs with more distant relatedness may be used, but the estimate may be adjusted and may be less robust.

### Simulated datasets

It is easier to explain our decisions made for method development using simulated data. We simulated all autosomal chromosomes using randomly selected individuals from the UK Biobank as the founder population. 1000 unrelated individuals from the UK Biobank [6] were selected and the population size at each generation was kept at 1000. The genetic data of the last four generations and their relationships were extracted for benchmarking (**S1 Table**). Additionally, four more datasets were generated where founder populations were selected only from individuals with the self-reported ethnic background as 1) African, 2) British, 3) African and British, and 4) British and African and Chinese.

For the simulation, the cross-overs for each chromosome were calculated using Poisson distribution (*λ* = L /50), where L is the chromosome length in cM. The genetic mapping file from deCode [22] was used for genetic mapping and mating patterns follow [23]. We randomly set up 500 non-overlapping couples and there was a 0.2 chance that a female may have a child with a different male individual randomly selected from the same generation. We also introduced phasing errors, genotyping errors with varying genotyping/phasing error rates, in a range that is typical of a modern biobank-scale, to verify the robustness of RAFFI (see **Results** section). We computed precision and recall for each degree of relatedness. Precisions is defined as the ratio of the correctly inferred degree of relatedness of pairs, and recall is defined as the ratio of recovered relatedness pairs for each degree:

$$recall=TP_{i}/P_{i}\;\mathrm{and}$$

$$precision=TP_{i}/({TP}_{i}+FP_{i}),$$

where *TP*_*i*_ denotes the correctly detected pairs for the i-th degree, *P*_*i*_ the total number of pairs in the degree i, and *FP*_*i*_ the falsely detected pairs in the i-th degree. Additionally, we computed the F1 value as the harmonic mean of recall and precision [24]:

F1=2*recall*precision/(recall+precision).

### UK Biobank dataset

The phased genotype data of the UK Biobank (version 2) data [6] comprising 487,409 participants and 658,720 sites were extracted. The majority of participants in the UKBB are of British descent, however, the data contain other ethnic backgrounds and also admixture populations, providing the option to simulate diverse populations. The genetic maps from deCODE for hg38 [22] were downloaded and lifted over to hg19 using the liftOver tool [25]. The longest monotonically increasing subset of the sites in each chromosome of hg19 was selected and subsequently interpolated to obtain the genetic locations of the available sites in the UKBB. Two subsets of UKBB were extracted, splitting the entire panel into individuals with British or non-British ethnic backgrounds (using Data-Field 21000). RAFFI was run on all three subsets: all UK participants, individuals with self-reported British ethnicity, and individuals from any other ethnic background.

### Software

RAFFI is freely available for use at https://github.com/ZhiGroup/RAFFI.

## Results

### Benchmarking using simulated data

**Fig 3:**
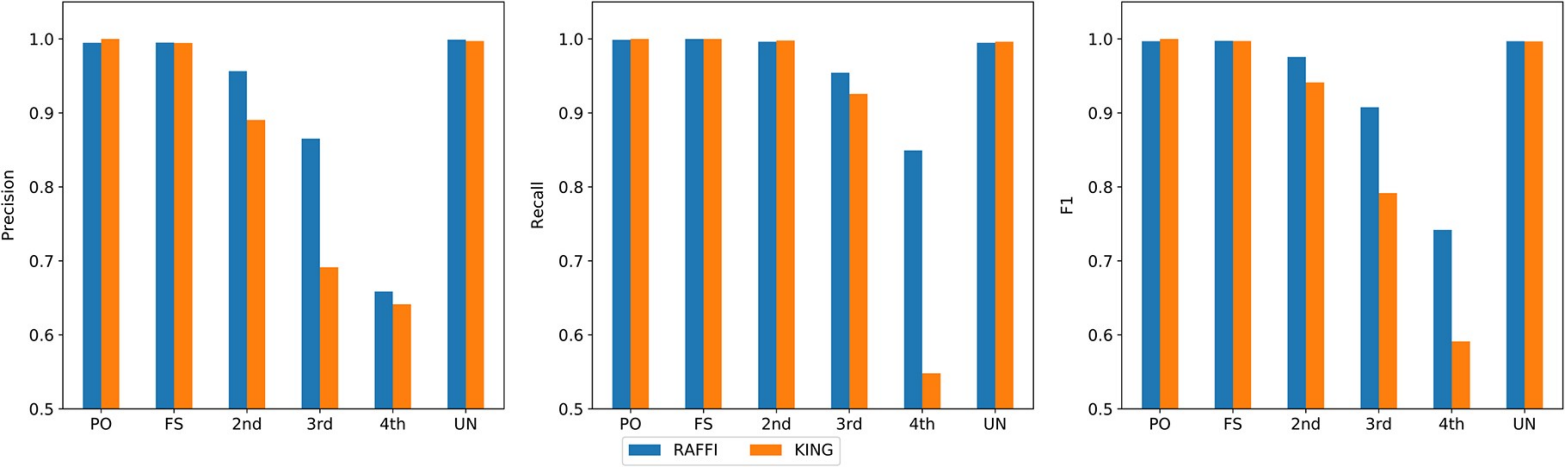
Comparison of results between RAFFI and KING in the simulated data. RAFFI shows higher precision and recall values for more distant relatives such as 3^rd^ or 4^th^ degrees of relatedness.

We benchmarked our approach RAFFI and KING using simulated data with varying phasing/genotyping errors, different marker densities, and diverse populations. The goal was to evaluate and demonstrate the robustness of our approach against different or misspecification of phasing, genotyping error rates, different marker densities, and the performance in different populations. We calculated precision and recall for the degrees of relatedness up to the 4^th^ degree. Any two individuals with a degree of relatedness 5 or more distant were considered as unrelated. Table 3 shows the inferred relationships up to 4^th^ degree for RAFFI and KING in the simulated data without any added genotyping or phasing errors. The F1 measures for both KING and RAFFI are high for up to the 2^nd^ degree while the difference becomes more distinct for the 3^rd^ and 4^th^ degrees (**Fig 3**). The percentage of falsely detected 3^rd^ degree relatives in KING is higher than RAFFI which is reflected in the precision values. KING overestimated proportionately more relatives as 3^rd^ degree. The gap between the precision results of KING and RAFFI decreases for the 4^th^ degree. On the other hand, the recalled pairs are significantly lower for 4^th^ degree values compared to RAFFI.

**Table 3:** Comparison of results of RAFFI and KING in the simulated data.

		KING						
		MZ	PO	FS	2nd	3rd	4th	UN
**RAFFI**	**MZ**	0	0	0	0	0	0	0
	**PO**	0	5993	3	28	0	0	0
	**FS**	0	7	4548	0	0	0	0
	**2nd**	0	0	5	25927	21	0	12
	**3rd**	0	0	0	1971	56619	116	1056
	**4th**	0	0	0	0	15833	71966	36765
	**UN**	0	0	0	0	0	10342	

Genotyping errors were implanted and also switch (phasing) errors were introduced in the simulated data. The genotyping errors range from 0.1 to 0.5% mimicking a realistic genotyping error rate in real data. Genotyping error rate is often low (e.g., 0.2% in WTCCC [26]) for human data. The selected range also contains the expected genotyping errors in the sequencing data which are usually higher. The phasing errors from 1 to 5 in every 20 cM on average were introduced. The UK Biobank data have an estimated long-range switch error of 1 in every 20 Mbps (~cM) [27]. The current biobank cohorts (e.g., UK Biobank) are estimated to have a switch error rate of 0.1–0.4% [21]. 0.1% switch error rate corresponds to almost 1 in every 40 cM and 0.4 switch error rate translates to almost 2 in every 20 cM. The performance of RAFFI and KING was also evaluated using different marker densities and in the presence of admixture populations. The precision, accuracy and F1 measures for all the experiments are available in S2 Appendix. For inferring relatives using KING (v.2.2) we chose the option—related—degree 4. This—related option is recommended by the authors when dealing with biobank-level datasets. This option computes the kinship values first and filters out pairs according to the ‘-degree’ option. After selecting the potential candidates, it estimates IBD segments among the potential pairs and subsequently infers relatedness. As a result, it should be more efficient in terms of run time compared to—ibd-seg option while having higher accuracy compared to—kinship option. The—ibd-seg option was also three times slower than the option—related—degree 4 in our simulation and thus not included in our comparison.

### Robustness against misspecification of genotyping error

**Fig 4:**
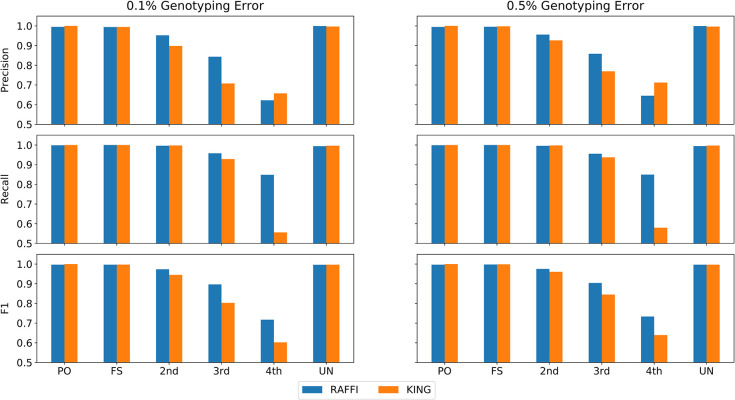
Robustness of relatedness inference against misspecification of genotyping error rate. Precision, Recall, and F1 values for RAFFI and KING using datasets with increasing genotyping error rates while a genotyping error of 0.1% was expected.

We assumed a genotyping error of 0.1%, estimated the parameters based on the expected error rate for RaPID, and searched for the relatives up to the 4^th^ degree. Note that RaPID needs an estimation of the genotyping error rate (which is usually between 0.1 to 0.5%) to estimate the parameters for IBD detection. To show the robustness of RAFFI against misspecification of genotyping error, we assumed a fixed genotyping error and ran the program with the same parameters on different panels with various genotyping error rates (from 0.1 to 0.5% with a step size of 0.1). The precision and recall of RAFFI remain high with increasing genotyping errors as shown in **Figs 4** and S2. F1 values (harmonic mean of precision and recall) remain constantly higher compared to KING. KING shows a slightly higher precision for the 4^th^ degree while the recall values are significantly lower (20–30%). We noticed that KING outputs different results based on the given maximum degree of relatedness. Apparently, KING discards a large set of pairs in the first step to avoid all pairwise comparisons. As a result, the recall will be lower but the precision will be slightly higher compared to RAFFI for 4^th^ degree. We benchmarked IBDkin [28] another IBD-based detection method, using different genotype errors. IBDkin reports up to 3^rd^ degree of relatedness and we had to limit our comparison up to 3^rd^ degree. While IBDkin shows high precision and recall values for a low genotyping error rate panel (0.1%), it does not guarantee robustness against higher genotyping errors (see **S3 Fig**).

### Robustness against phasing error

**Fig 5:**
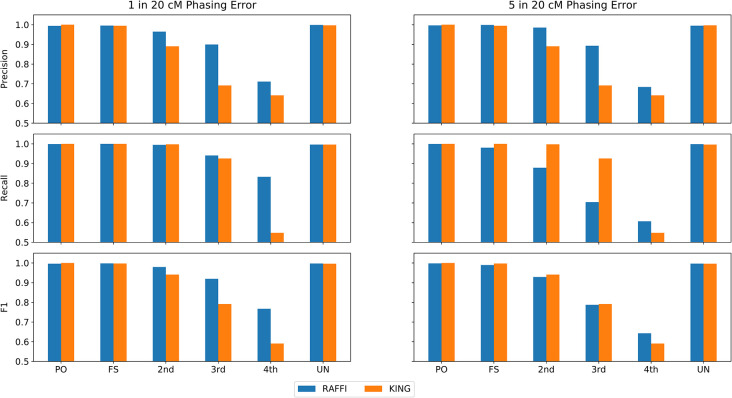
Robustness of relatedness inference against phasing error. Precision and Recall and F1 values for RAFFI and KING with different phasing (switch) error rates. While KING is expected to be immune to phasing error, RAFFI also shows robustness to phasing error after adjustment of kinship coefficients.

Switch errors in addition to genotyping errors may reduce the detection power of IBD detection methods working with phased data. RaPID has been shown to maintain high detection power for the target length of 5 cM with 1 phasing error in 20 cM (~20 Mbps) on average. While the current phasing approaches may not result in an abundance of phasing errors, the presence of phasing errors in haplotypes is currently inevitable without trio information. The availability of large reference panels, however, can increase the phasing quality significantly. For example, it is expected to observe a major switch error of about 1 in every 20 cM [20,21] in the UK Biobank. Any switch error may potentially decrease the length of detected IBD segments using haplotype data. Further increase of phasing errors, however, may result in further reduction of detection power. The adjustment of threshold values may be more crucial for panels with high phasing errors. We added switch errors in the simulated data from 1 to 5 successively and searched for the relatives up to the 4^th^ degree of relatedness. Again, we assumed a genotyping error of 0.1% for estimating the parameters of RaPID. **Fig 5** shows the precision and recall results in panels with different phasing errors with 1 and 5 switch errors in every 20 cM on average. Different phasing errors from 1 to 5 in every 20 cM are shown in **S4 Fig**. Both precision and accuracy values of RAFFI are higher compared to KING even with the panels with a high number of switches. Please note that methods like KING leverage the unphased genotype data and phasing errors should not impact the results of KING at all.

### Robustness against phasing and genotyping error

**Fig 6:**
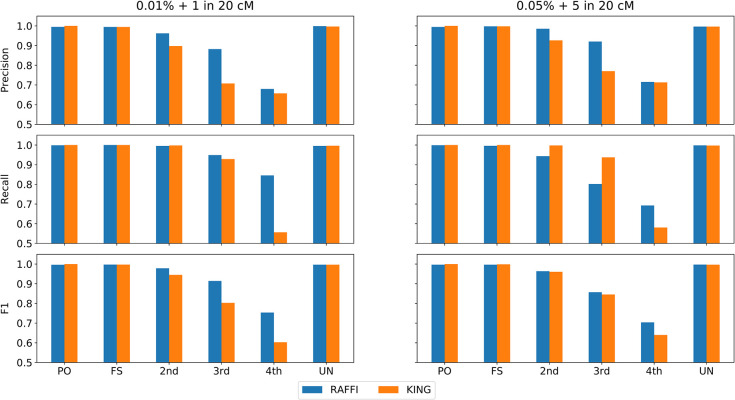
Precision and Recall and F1 values for RAFFI and KING with different phasing (switch) and genotyping error rates. The adjustment of kinship coefficients accounts for both phasing and misspecification of genotyping error at the same time.

The objective was to investigate whether RAFFI can handle both the misspecification of genotyping errors and switch errors. More specifically, whether the kinship coefficient adjustment approach remains robust with introducing genotyping and phasing errors at the same time. We added switch errors in the simulated data from 1 to 5 within 20 cM successively. We assumed genotyping errors at a rate of 0.1% and estimated the parameters for RaPID accordingly while the actual genotyping error rate varied from 0.1 to 0.5%. The benchmarking results show that RAFFI is robust against both phasing and genotyping errors (**Figs 6** and S5), a necessary feature to be able to handle real data accurately. An extensive number of genotyping errors (0.5%) and phasing errors (5 per 20 Mbps) will result in lower recall for 3^rd^ and 4^th^ degrees, while the precision is high, and the total F1 measure remains higher compared to KING. Due to the minimum cut-off of 5 cM (and an extensive number of mutations), some pairs of 4^th^ degrees may not share any IBD segments. A subset of 3^rd^ degree pairs has also been classified as 4^th^ degree relatives which translates into lower recall for 3^rd^ degree relatives.

### Robustness over different marker densities

**Fig 7:**
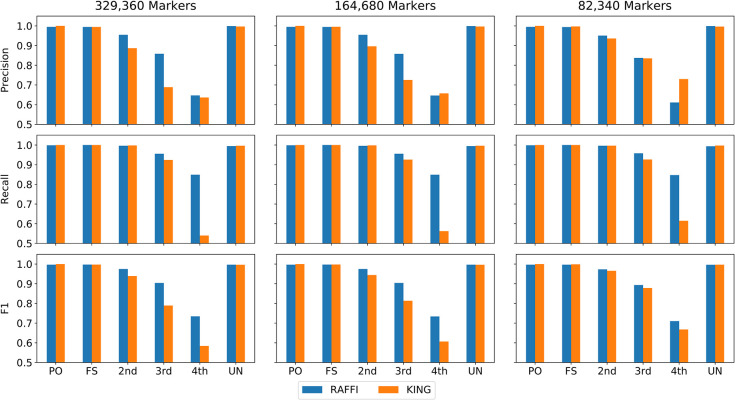
Precision, Recall, and F1 values for RAFFI and KING using different maker densities. Reducing the original maker density by half and ¼ will not impact the results of RAFFI.

To benchmark the robustness of RAFFI against varying marker density, we thinned the genotype data by subsampling. Low marker density can reduce the accuracy of detected IBD segments which may affect the results of relatedness inference using IBD segments. The marker density of the UK Biobank is already not very high. However, some data sets may have an even lower density than UK Biobank. We then selected ½ and ¼ and ⅛ of the markers in the simulated data and ran RAFFI and KING. The precision and recall of RAFFI remain high with reducing the marker density in the simulated data as shown in **Fig 7**.

### Robustness in admixture populations

**Fig 8:**
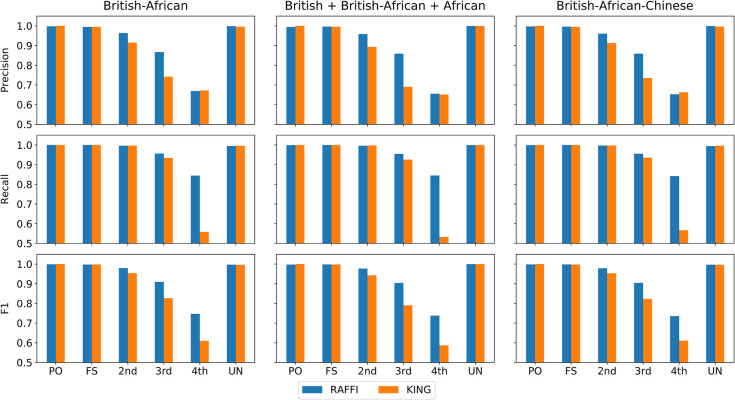
Precision, Recall, and F1 values for RAFFI and KING using admixture populations. RAFFI demonstrates higher precision and recall especially in the heterogeneous panel containing British, British-African, and African people.

In order to verify the robustness of RAFFI against different population structures, we simulated admixture populations using different ethnic groups from the UKBB. A group of 1000 individuals from each of the British, African, and Chinese individuals in the UKBB were selected randomly as founder populations. Five panels were created by using each of the populations and also a combination of two (British, African) and all three populations (British, African, and Chinese) as founder populations. **Fig 8** shows the results for 1) British-African, 2) a combination of British, British-African, and African, and 3) British-African-Chinese admix populations. RAFFI demonstrates high precision and recall, while the recall value of KING is low especially for the 4^th^ degree of relatedness. The advantage of using RAFFI is more obvious in heterogeneous panels containing admixture populations and also individuals without recent admixture events (from both populations) as shown in the middle panel.

### Inferring related individuals in UK Biobank

We ran both KING and RAFFI on UK Biobank data and compared the run time and detected pairs of relatives. RAFFI and KING are consistent for very close relatives (monozygotic twins and first degree), however, the discrepancies become more obvious for more distant relatives. Table 4 shows the comparison of relatives detected by KING and RAFFI using individuals with (self-reported) British ethnicity in UK Biobank. The detected pairs among very close relatives (such as Parent-offspring, Full-sibling) are very consistent. For 4^th^ degree relatives, however, RAFFI outputs significantly more pairs compared to KING. We speculate that KING uses aggressive filtering (based on the given maximum degree) which might result in discarding a significant portion of true 4^th^ degree relatives. The results for all UK participants and also non-British people are summarized in **S2 and S3 Tables**. Some pairs that have been reported by RAFFI as 2^nd^ or 3^rd^ degree have been classified among non-British people as closer relatives by KING which could be due to overestimation of KING [29] or extensive phasing errors for minorities in the UK Biobank. The latter would cause lower detection power of IBD segments only in a subset of data which may not be addressed by adjusting the kinship coefficients using the average of expected coefficients. The total run time for RAFFI (IBD detection and inference of relatives) was almost 18 times faster than KING using UK Biobank data on a single CPU. The run times of RaPID and KING in simulated data and UK Biobank are available in **Table 5**.

**Table 4:** Comparison of results of RAFFI and KING using British individuals in UK Biobank.

		KING						
		MZ	PO	FS	2nd	3rd	4th	unrelated
**RAFFI**	**MZ**	164	0	0	0	0	0	0
	**PO**	0	5669	0	1	0	0	0
	**FS**	0	0	20273	5	0	0	0
	**2nd**	0	0	11	9820	25	0	14
	**3rd**	0	0	0	1346	54762	177	113
	**4th**	0	0	0	0	8899	63891	19775
	**unrelated**	0	0	0	0	1	15804	0

**Table 5:** Run time comparison between RAFFI (excluding the phasing time) and KING in simulated and UK Biobank data using all participants.

#Cores	Tool	Dataset	Wall Time*
1	RAFFI	Simulation	00:27:14
		UKB	~ 5 days
	KING	Simulation	00:06:58
		UKB	~ 90 days
24	RAFFI	Simulation	00:02:51
		UKB	~ 15 h
	KING	Simulation	00:00:43
		UK Biobank	~ 4 days

* The experiments were run on a server with Intel(R) Xeon(R) Silver 4116 CPU @ 2.10GHz.

### Run time and memory usage

The run time and memory efficiency of RAFFI are more obvious when it is applied on large panels comprising hundreds of thousands of individuals. The current implementation of RAFFI allows for multi-threading. IBD calling of each or multiple chromosomes will be performed in a separate thread using RaPID based on the given number of threads. The second step which includes the inference of relatedness can also be run in parallel where each thread is assigned a subset of potentially related pairs to analyze. **S4 Table** shows the run time and memory peak usage of RAFFI and KING using simulated data and UK Biobank. The time complexity of RAFFI is mainly impacted by the detected IBD segments in the panel. Despite the efficient method for computing kinship in KING, it may not grow linearly with the sample size which results in significantly longer run time. Moreover, RAFFI does not require a lot of memory (see **S4 Table**). KING loads the genotype panel into memory which enables fast access and computation of kinship values. On the other hand, it will require extensive memory for large panels. RAFFI does not require to load entire genotype data in the memory at any step. The peak memory usage of KING is 18.5 times more than RAFFI for the UK Biobank data (74 GB vs. 4 GB). The space complexity of RAFFI involving detection of IBD segments and inferring relatedness is *O*(*max*(*M*, *number of potential relatives*)), where *M* denotes the total number of samples and *number of potential relatives* denotes the number of individual pairs sharing an IBD segment.

## Discussions

We developed an efficient approach that leverages IBD segments to infer relatives in large biobank-scale cohorts. Our simulation results show that RAFFI is accurate up to the 4^th^ degree of relatedness using 5 cM IBD segments detected by RaPID. Moreover, it is robust against misspecification of genotyping error, phasing errors, and varying marker density. Using RaPID and adjusting the kinship coefficients resulted in higher precision/recalls compared to KING, especially for 3^rd^ and 4^th^ degrees. Both methods have high precision/recall values for relatives up to the 2^nd^ degree. Moreover, RaPID is able to call IBD segments in phased data without the pairwise comparison of individuals which makes it suitable for analysis of large cohorts. For large biobank-scale cohorts, phasing is often part of the standard processing. In that case, RAFFI will not incur the additional cost of phasing. If phasing is not available, non-IBD-based methods such as KING might be more appropriate.

During the revision of this manuscript, we noticed that IBDkin [28] is a recently developed method for fast relatedness inference using IBD information. Methodologically, both IBDkin and RAFFI use efficiently detected IBD segments first, and then post-process the IBD segments to make relatedness calls. RAFFI uses RaPID for IBD segment calls, which is more flexible making tradeoffs between run time, detection power, and accuracy. RAFFI optimizes the whole pipeline end-to-end even though the IBD segment detection, as an intermediate result, is not necessarily optimized for accuracy. IBDkin, on the other hand, is direct post-processing of hap-IBD outputs without joint optimization. Based on our new results, IBDkin appears to not be robust against genotyping errors (**S2 Fig**). Moreover, IBDkin is less scalable to large cohorts than RAFFI: For UK Biobank data, the run time of IBDkin is estimated to be 2–3 times faster than KING while RAFFI is 18x faster than KING. Moreover, 234.8 GB max memory was also reported for IBDkin compared to 4 GB for RAFFI (KING 74 GB).

We focus on IBD segments that are long enough (> 5 cM) which are unlikely to be false positives. Still, sharing IBD segments may not directly indicate close relationships. Populations may have varying degrees of relatedness (inbreeding) and thus may have varying degrees of background IBD sharing [30]. In RAFFI we implicitly made the simplifying assumption that the background IBD level is zero, and thus we can reuse KING’s decision boundaries based on theoretical calculation. In future work, we may explore estimating the background IBD levels using a data-driven approach. Note that the background IBD levels should be much lower than the IBD levels of the full-siblings, and thus adjustment for phasing errors should be still valid in the presence of background IBD levels.

There is a growing trend in data-driven approaches for relatedness inference. This is because the real data are often blended with various artifacts, even though decision boundaries based on IBD1 and IBD2 are clear in theoretical settings. While we derived a data-driven approach for adjusting decision boundaries for RAFFI, we noticed that related work of [31] also adopted a data-driven approach for discerning various subtypes of 2^nd^ degree relatedness. We expect more data-driven approaches can be developed to make relatedness inference methods more practical.

Inferring relatedness based on the genetic similarity scores such as KING may result in overestimation in the presence of (recent) admixtures in the data set. We also observed a reduction in precision/recall of KING in the presence of admixture and multi-ethnic populations. To tackle this issue, some heuristics have been applied to filter out markers with MAF in an analysis of a subset of 459,777 individuals from the Million Veteran Project [29]. Haplotype based IBD detection methods, however, are robust to admixture and other population heterogeneities. RAFFI also will not be affected by the presence of admixture populations in the dataset.

The run time of RAFFI can also be further improved by optimization regarding I/O operations in RaPID. We used RaPID v.1.7 for this project which takes phased haplotypes in a compressed VCF format. Other data formats such as PLINK binary format (as used in KING) and the GDS format [32] would improve the run time of RaPID significantly.

While the IBD segments with a length of 5 cM or above were used, longer or shorter IBD length cutoff can be used depending on the situation. For example, longer IBD segments (e.g. 10 cM) may also be leveraged which will result in a short run time. In general, IBD segments of longer length can be detected faster using RaPID. For example, second cousins (4^th^ degree) would share at least an IBD segment with a length of 10 cM or above with a very high likelihood. If the data are well-phased then 10 cM cutoffs would be sufficient for inferring relatedness up to 4^th^ degree of relatedness.

One of the limitations of RAFFI is that the current implementation of RAFFI is only tested to distinguish degrees of relatedness up to 4^th^ degree and unrelated (5^th^ or more). A similar approach may be used to infer more distant relatives which are beyond the scope of this work. There are two major issues regarding the inference of distant relatives: Whether two individuals share an IBD segment at all with the given target length and are the detected IBD segments accurate. Very short exact matches may be due to IBS (Identity by State) rather than IBD. Moreover, the extensive number of short IBS/IBD segments may slow down the detection of IBD segments significantly due to extensive I/O operations. Investigation of these problems warrants further research.

Another limitation of RAFFI is that it is mainly designed for large scale cohorts of high quality human genotype data. RAFFI has indeed made assumptions about the sample size, inclusion of full-sibs, and quality of genotype data such as genotyping errors, phasing errors, and marker density. Extending RAFFI approach to other types of genotype data may be topics of future research.

## Supporting information

S1 Fig Computed kinship coefficients in a panel without any phasing/genotyping errors (Φ_1_) versus in a panel containing phasing errors (Φ_2_). (PNG)

S2 Fig Precision, Recall and F1 values for RAFFI and KING using datasets with increasing genotyping error rates from 0.1 to 0.5%. (PNG)

S3 Fig Precision, Recall and F1 values for RAFFI, IBDKin and KING using datasets with increasing genotyping error rates from 0.1 to 0.4%. (PNG)

S4 Fig Precision, Recall, and F1 values for RAFFI and KING with an increasing number of switch errors from 1 to 5 every 20 cM. (PNG)

S5 Fig Precision and Recall and F1 values for RAFFI and KING with different phasing (switch) and genotyping error rates. (PNG)

S1 Table Number of pairs for different degrees of relatedness in simulated data. (PDF)

S2 Table Comparison of results of RAFFI and KING using all participants in the UK Biobank data. (PDF)

S3 Table Comparison of results of RAFFI and KING using non-British people in the UK Biobank data. (PDF)

S4 Table Run time and peak memory comparison of RAFFI and KING. (PDF)

S1 Appendix Pseudocode for adjustment of kinship coefficients. (PDF)

S2 Appendix Precision, Recall and F1 values for RAFFI, KING and IBDkin for all the benchmarks using simulated data. (XLSX)
