# Supplementary figures and images for: RAFFI: Accurate and fast familial relationship inference in large scale biobank studies using RaPID
Source: PLoS Genet. 2021 Jan 21;17(1):e1009315. doi: 10.1371/journal.pgen.1009315 (PMC7853505; doi:10.1371/journal.pgen.1009315)

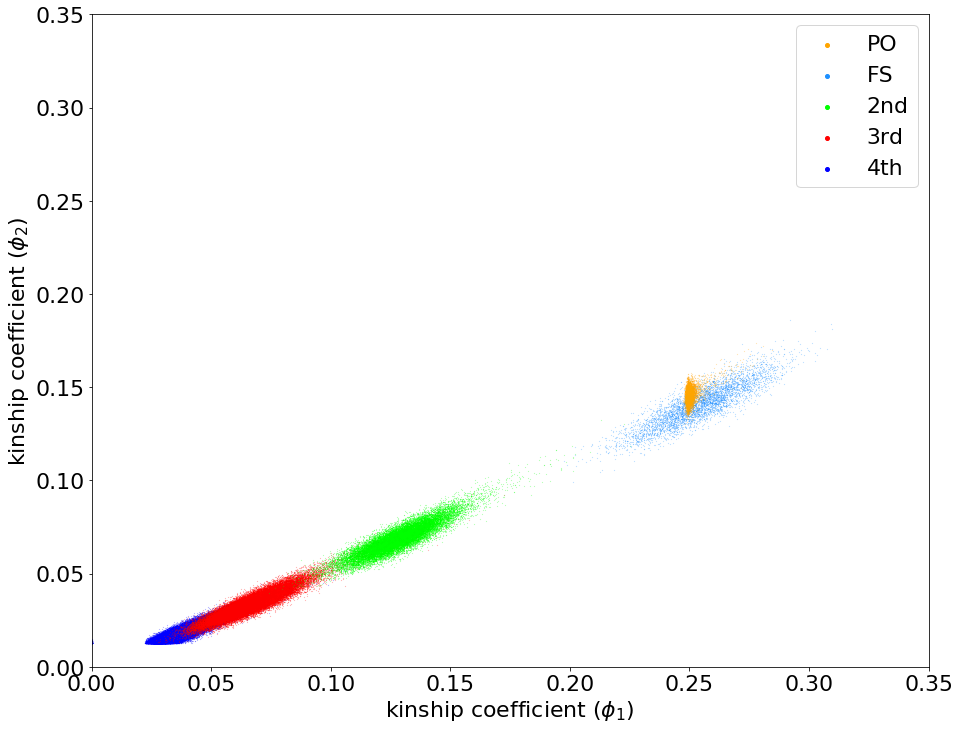

Supplement: S1 Fig — (PNG) [file pgen.1009315.s001.png]

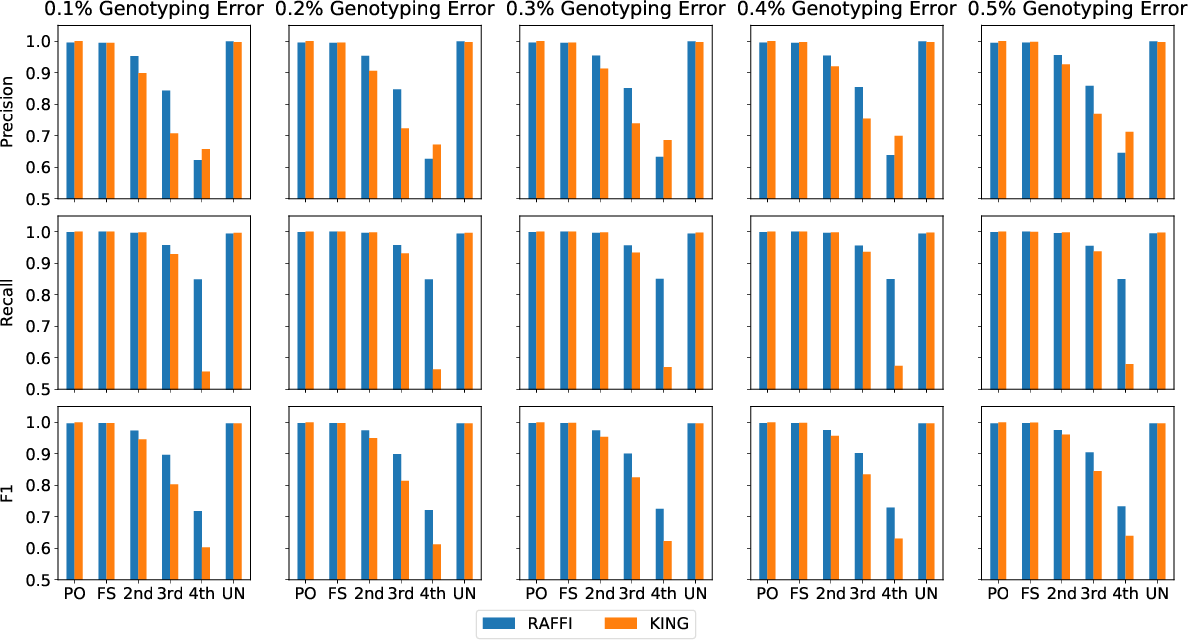

Supplement: S2 Fig — (PNG) [file pgen.1009315.s002.png]

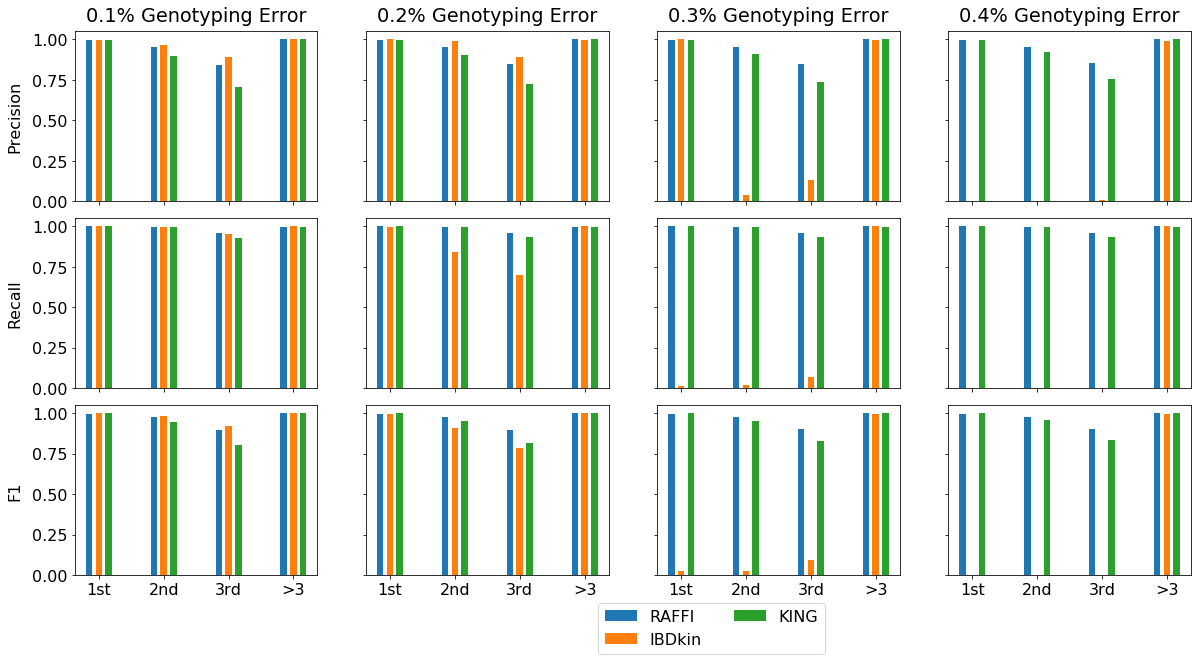

Supplement: S3 Fig — (PNG) [file pgen.1009315.s003.png]

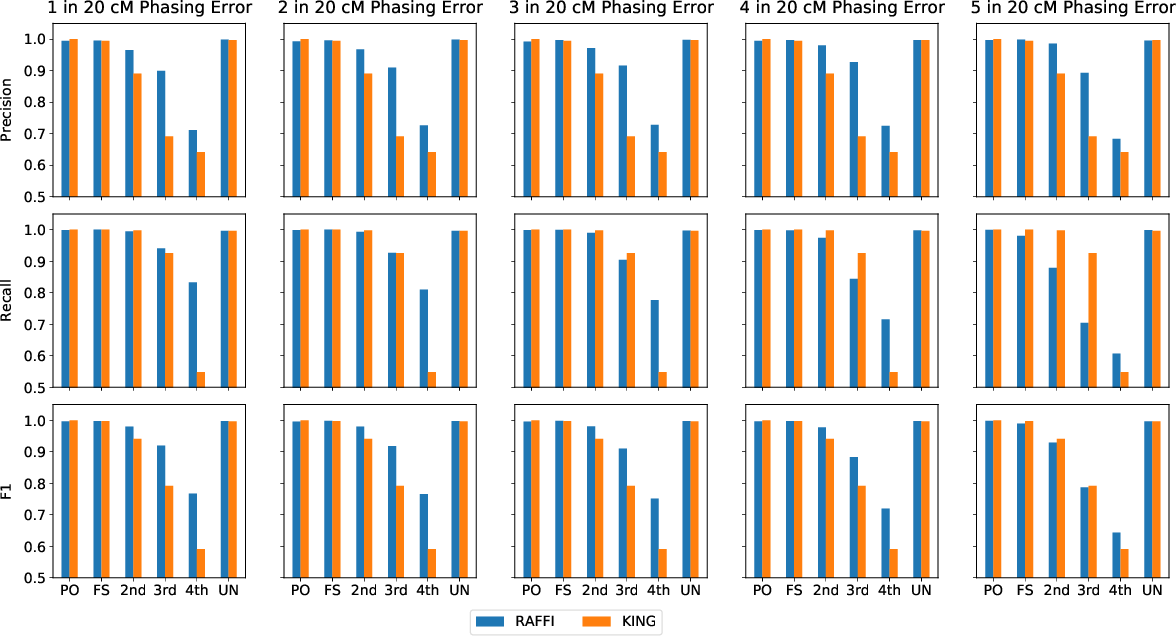

Supplement: S4 Fig — (PNG) [file pgen.1009315.s004.png]

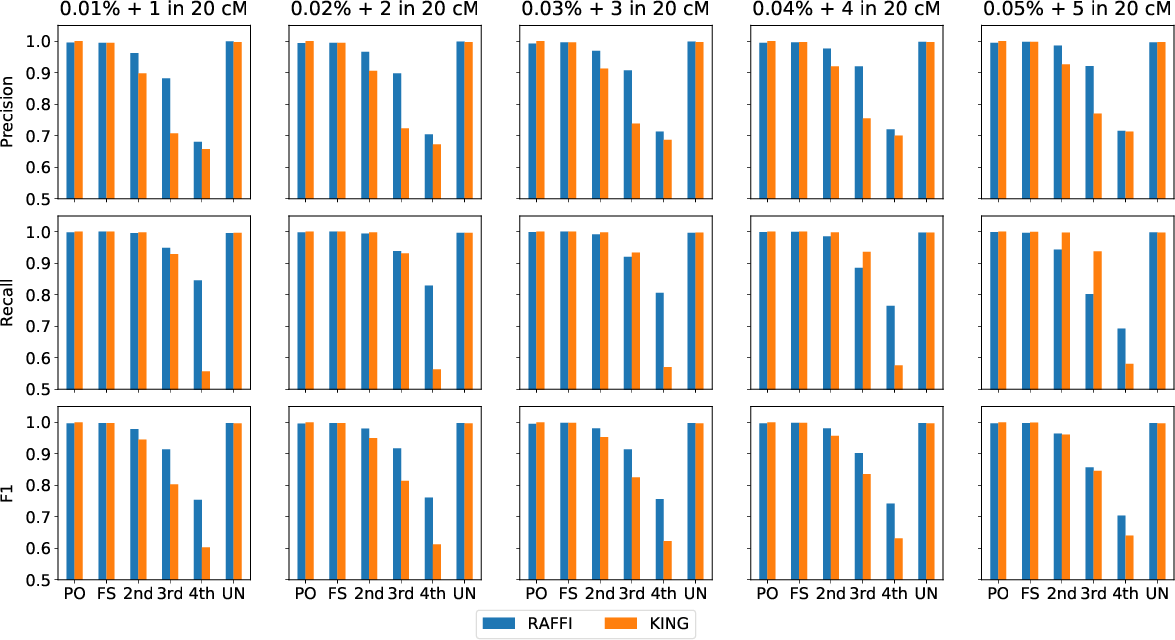

Supplement: S5 Fig — (PNG) [file pgen.1009315.s005.png]
