# Supplementary material for: RAFFI: Accurate and fast familial relationship inference in large scale biobank studies using RaPID
Source: PLoS Genet. 2021 Jan 21;17(1):e1009315. doi: 10.1371/journal.pgen.1009315 (PMC7853505; doi:10.1371/journal.pgen.1009315)
Supplement: S1 Table — (PDF) [file pgen.1009315.s006.pdf]

S1 Table: Number of pairs for different degrees of relatedness in simulated data.

Relationship	Number of Pairs
**Parent/Offspring**	6000
**Full sibling**	4531
**2nd degree**	24,926
**3rd degree**	54,130
**4th degree**	96,477
**Unrelated (5th degree and more)**	7,811,936
**Total**	**7,998,000**
