# Supplementary material for: RAFFI: Accurate and fast familial relationship inference in large scale biobank studies using RaPID
Source: PLoS Genet. 2021 Jan 21;17(1):e1009315. doi: 10.1371/journal.pgen.1009315 (PMC7853505; doi:10.1371/journal.pgen.1009315)
Supplement: S2 Table — (PDF) [file pgen.1009315.s007.pdf]

S2 Table: Comparison of results of RAFFI and KING using all participants in the UK Biobank data.

		KING						
		**MZ**	**PO**	**FS**	**2nd**	**3rd**	**4th**	**unrelated**
**RAFFI**	**MZ**	179	0	0	0	0	0	0
	**PO**	0	6184	0	1	0	0	0
	**FS**	0	2	22339	5	9	0	
	**2nd**	0	62	299	10875	32	0	16
	**3rd**	0	22	24	1585	59363	207	168
	**4th**	0	4	1	65	9780	68212	22519
	**unrelated**	0	3	0	19	175	17728	
