# Supplementary material for: RAFFI: Accurate and fast familial relationship inference in large scale biobank studies using RaPID
Source: PLoS Genet. 2021 Jan 21;17(1):e1009315. doi: 10.1371/journal.pgen.1009315 (PMC7853505; doi:10.1371/journal.pgen.1009315)
Supplement: S3 Table — (PDF) [file pgen.1009315.s008.pdf]

S3 Table: Comparison of results of RAFFI and KING using non-British people in the UK Biobank data.

		KING						
		**MZ**	**PO**	**FS**	**2nd**	**3rd**	**4th**	**unrelated**
**RAFFI**	**MZ**	12	0	1	0	0	0	0
	**PO**	0	202	0	6	0	0	0
	**FS**	0	0	1039	0	0	0	0
	**2nd**	0	42	192	437	30	0	3
	**3rd**	0	10	1	119	1331	30	96
	**4th**	0	2	0	51	329	789	1478
	**unrelated**	0	0	0	5	101	895	0
