# Supplementary material for: RAFFI: Accurate and fast familial relationship inference in large scale biobank studies using RaPID
Source: PLoS Genet. 2021 Jan 21;17(1):e1009315. doi: 10.1371/journal.pgen.1009315 (PMC7853505; doi:10.1371/journal.pgen.1009315)
Supplement: S4 Table — (PDF) [file pgen.1009315.s009.pdf]

S4 Table: Run time and peak memory comparison of RAFFI and KING.

Core	#samples	Tool	Wall time	Peak Memory
**1**	4000	RAFFI	00:27:14	248 MB
		KING	00:06:58	924 MB
	487409	RAFFI	~5 days	~4 GB
		KING	~90 days	~74 GB
**24**	4000	RAFFI	00:02:51	694 MB
		KING	00:00:43	942 MB
	487409	RAFFI	~15 h	~4 GB
		KING	~4 Days	~74 GB
