# Supplementary material for: RAFFI: Accurate and fast familial relationship inference in large scale biobank studies using RaPID
Source: PLoS Genet. 2021 Jan 21;17(1):e1009315. doi: 10.1371/journal.pgen.1009315 (PMC7853505; doi:10.1371/journal.pgen.1009315)
Supplement: S1 Appendix — (PDF) [file pgen.1009315.s010.pdf]

S1 Appendix: Pseudocode for adjustment of kinship coefficients

The following pseudo-code illustrates the adjustment of kinship coefficients using detected full-sibling pairs iteratively. The objective of the iterations is to capture a sufficient amount of full-sibling pairs (1000 by default) which are then used to estimate the adjustment factor. $\Phi^{\alpha i}(j, k)$ represents the kinship coefficient between the individuals *j* and *k* at the *i*-iteration using the adjustment factor α_i. $\pi_2^{\alpha i}(j, k)$ denotes the IBD2 segment proportion between the individuals *j* and *k* at the *i*-iteration. $F(\alpha_i)$ is the number of full-sibling pairs at the *i*-th iteration.

```
i = 0, min_num_fs = 50, prev_adj_num_fs = 0, max_num_fs = 1000, Φmin^αi=0.1

For j, k in S:  // S is the set of pairs sharing IBD segments between two pairs j and k
        Compute Φ^αi (j, k), π2^αi (j, k)
        If (Φ^αi > Φmin^αi AND (|F (αi)| < min_num_fs OR |F (αi)| - prev_adj_num_fs < min_adj_intervals) :

                Infer relationship of j, k and store it temporarily

        Else if |F (αi)| >= min_num_fs AND |F (αi)| - prev_adj_num_fs > min_adj_intervals:

                prev_adj_num_fs = |F (αi)|

                compute αi+1

                i = i +1

                Update the relatedness (boundaries) using αi

        If |F (αi)| > max_num_fs:

                Return αi
```

Pseudo-code for adjustment of kinship coefficients iteratively.
